# Supplementary figures and images for: G-quadruplexes formed by Varicella-Zoster virus reiteration sequences suppress expression of glycoprotein C and regulate viral cell-to-cell spread
Source: PLoS Pathog. 2023 Jan 11;19(1):e1011095. doi: 10.1371/journal.ppat.1011095 (PMC9873165; doi:10.1371/journal.ppat.1011095)

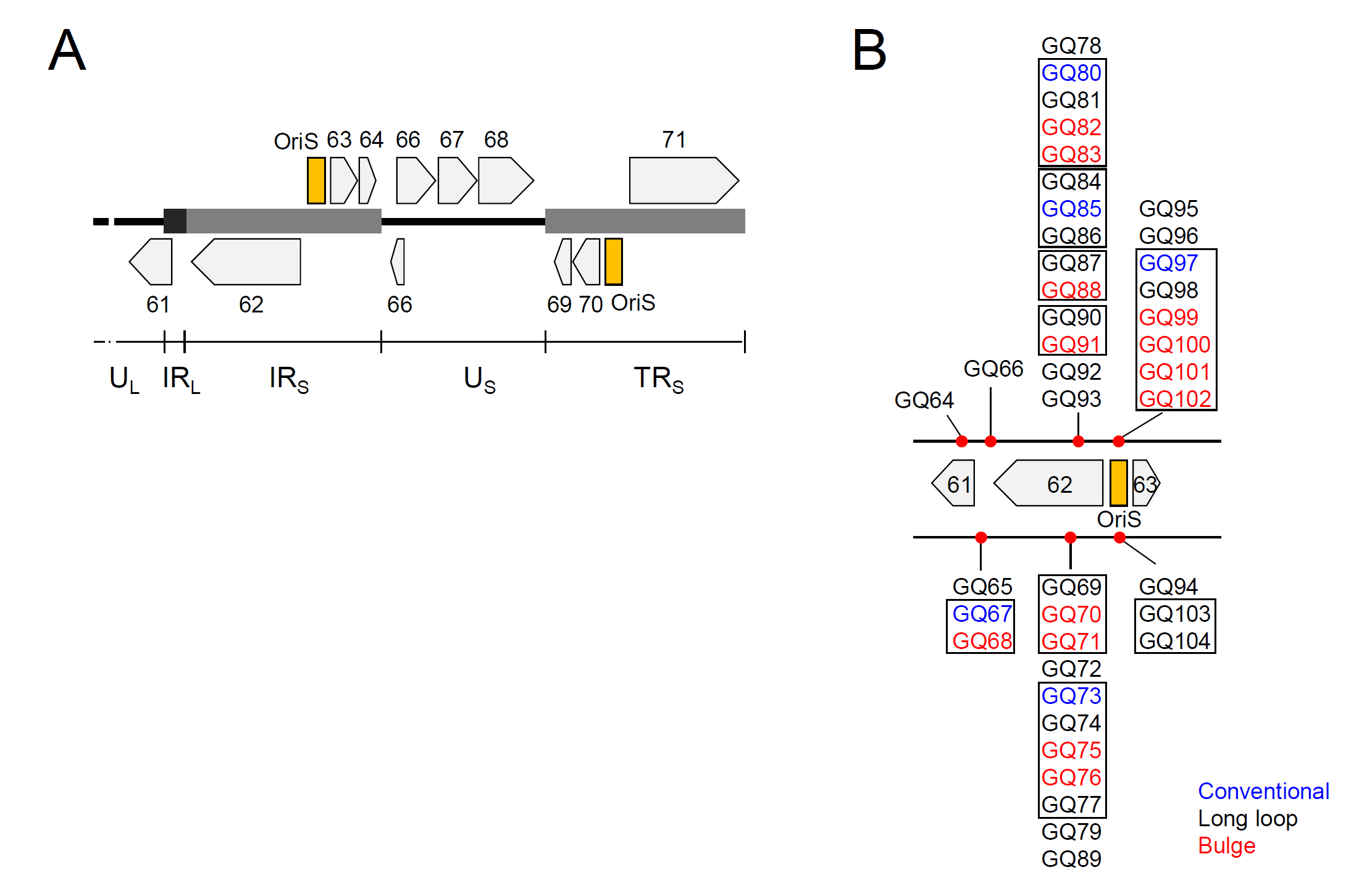

Supplement: S1 Fig — (A) The positions of ORFs and OriS in the IRL, IRS, US and TRS regions in the VZV genome are shown. (B) The G4 motifs (GQ64 to GQ104) found in IRL and IRS are indicated on the top and bottom strands of the genome. Similar G4 motifs with overlapping sequences are clustered in a box. The types of G4 (conventional, long-loop, and bulged) are indicated with different colors. (TIF) [file ppat.1011095.s003.tif]

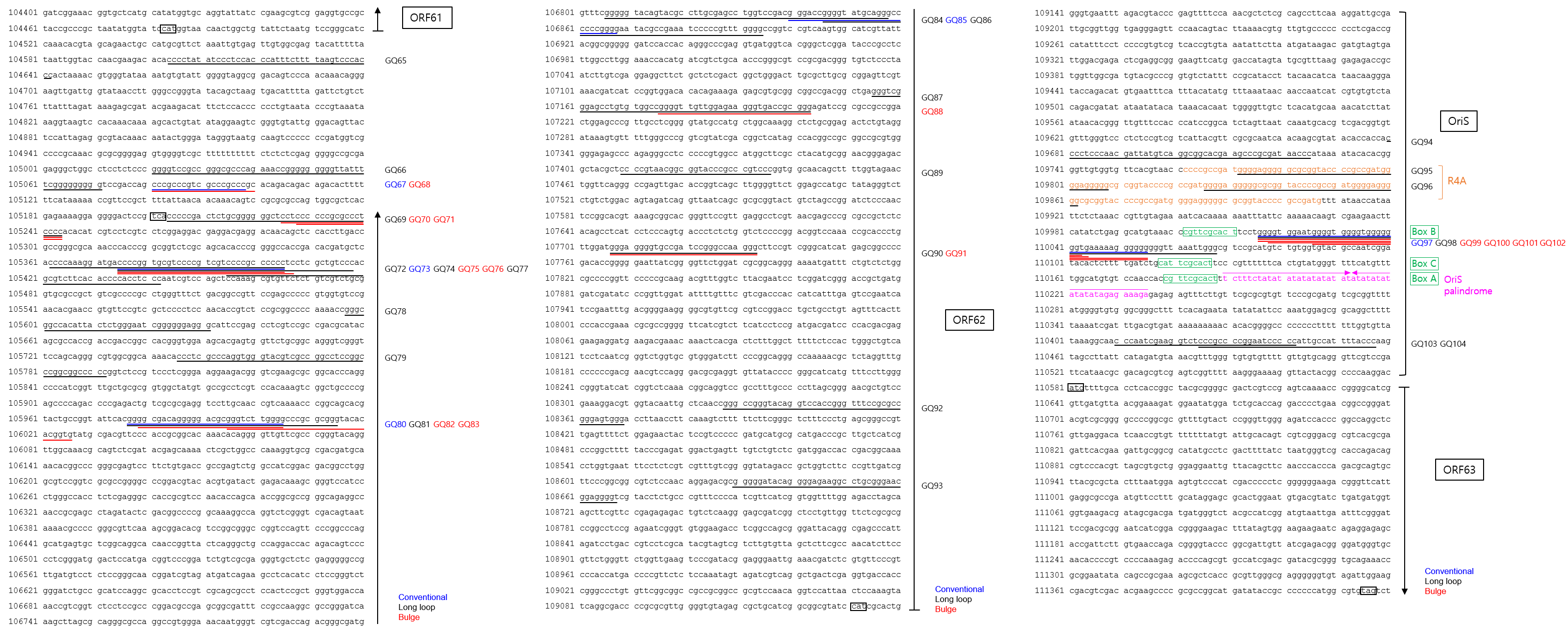

Supplement: S2 Fig — The G4 motifs (GQ65 to GQ104) found in IRS are indicated below the genome sequence of Dumas. The G4 motifs predicted for conventional, long-loop, and bulged G4s are indicated as blue, black, and red lines, respectively. The positions of ORF61, ORF62, OriS, and ORF63 are indicated. In OriS, the locations of the R4A reiteration sequence, Boxes A, B, and C, and palindrome sequence are indicated. (TIF) [file ppat.1011095.s004.tif]

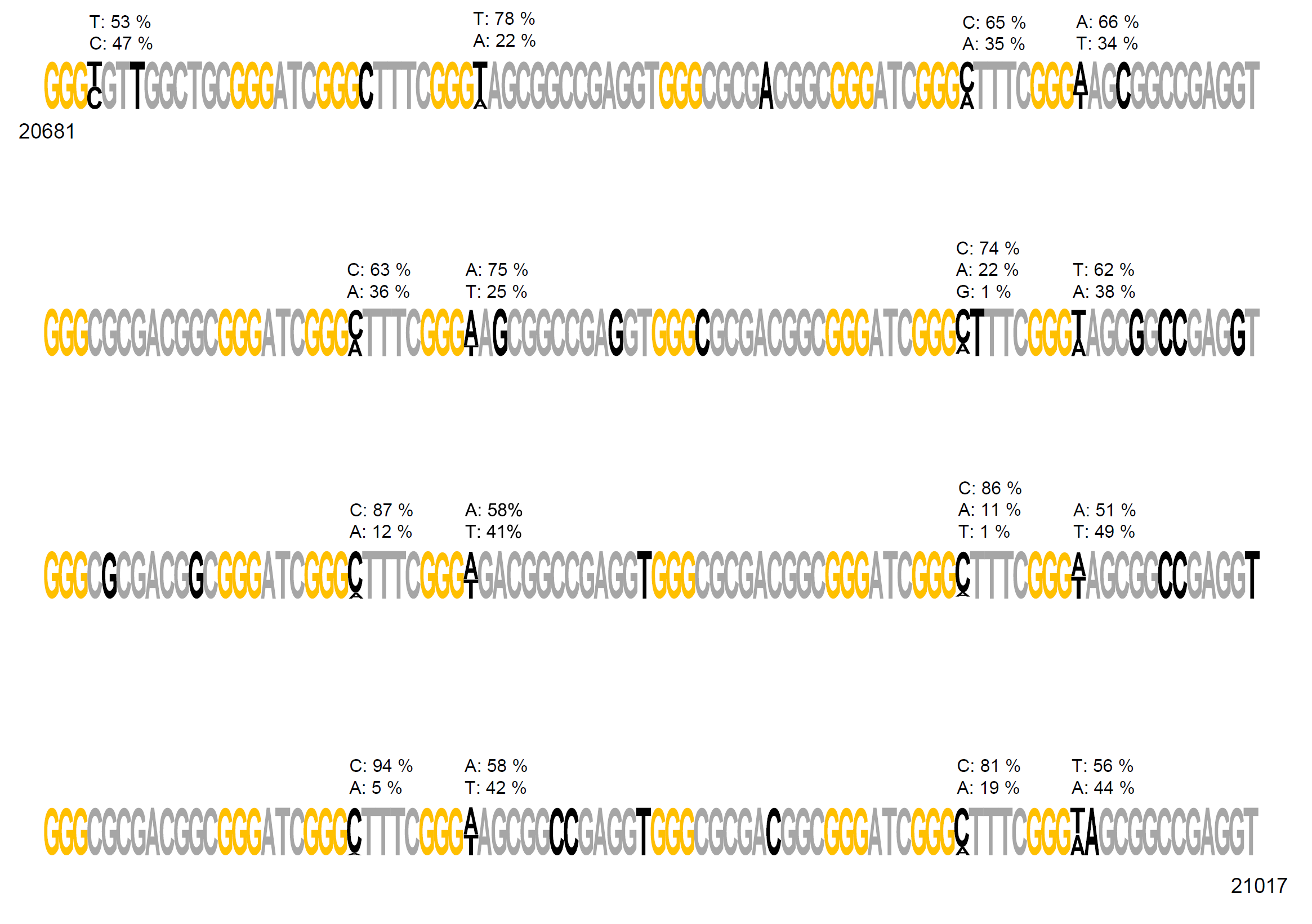

Supplement: S3 Fig — Multiple nucleotide sequence alignment of the R2 reiteration regions in ORF14 of 141 VZV strains was performed using MAFFT [78,79]. R2 of the DUMAS strain (from 20,681 to 21,017 in the genome) was used as the reference sequence. Graphical representation of R2 variation indicates preserved G-runs (3 Gs) in orange, other preserved sequences in gray, and sequences with variations in black letters. Nucleotide sequences with <1% variations are represented by a single black letter. The degree of variation in nucleotides is indicated as a percentage. (TIF) [file ppat.1011095.s005.tif]

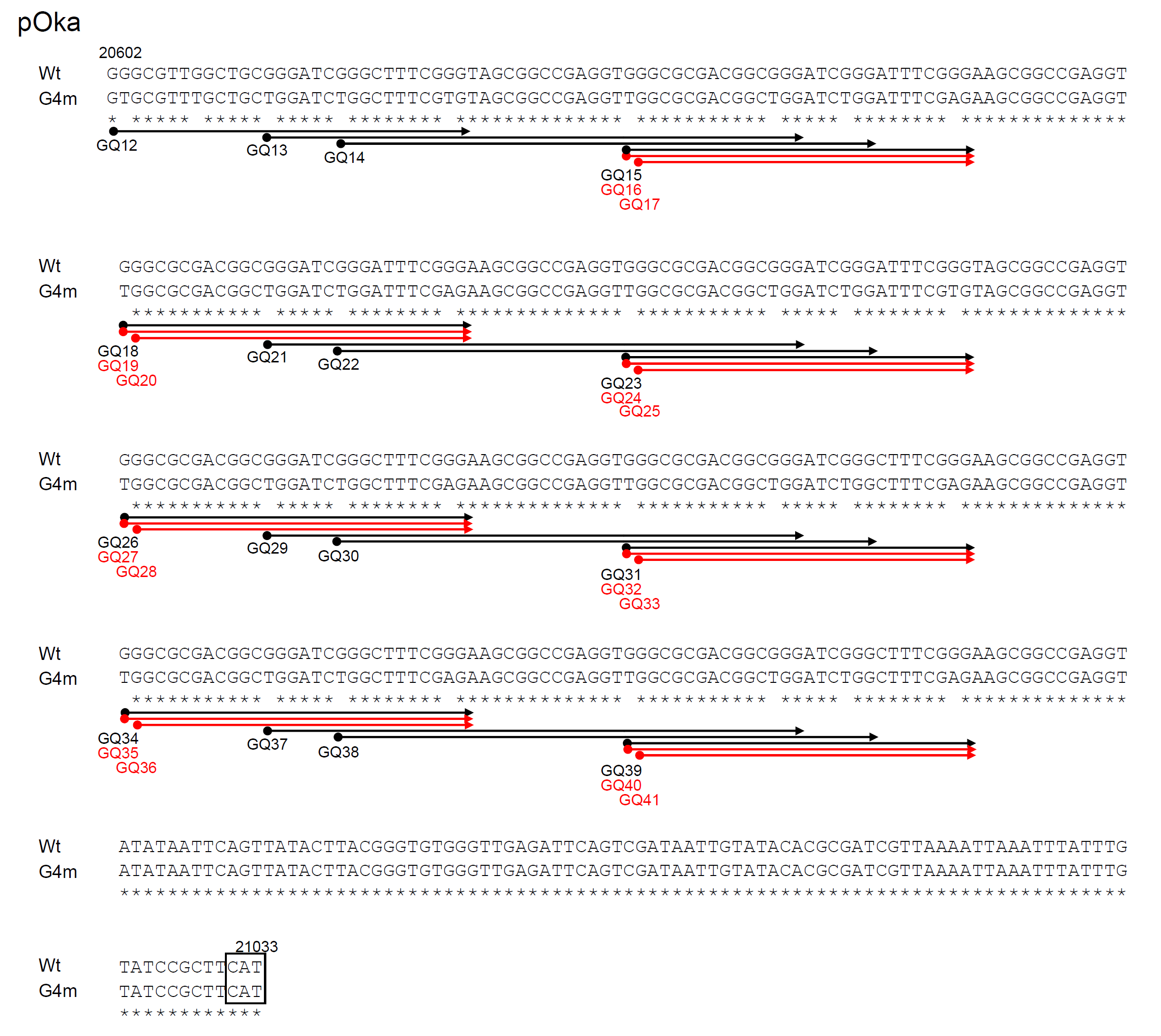

Supplement: S4 Fig — Alignment of the top strand sequences for part of ORF14 from 20602 to 21033 (in pOka) with its corresponding G4-disrupted mutant (G4m) sequences is shown. The identical sequences are denoted as *. The positions of G4 motifs (GQ12 to GQ41) in pOka are indicated below the sequences. Black arrows, long-looped G4; red arrows, bulged G4. The sequences corresponding to the translation initiation codon for gC are indicated in the box. (TIF) [file ppat.1011095.s006.tif]

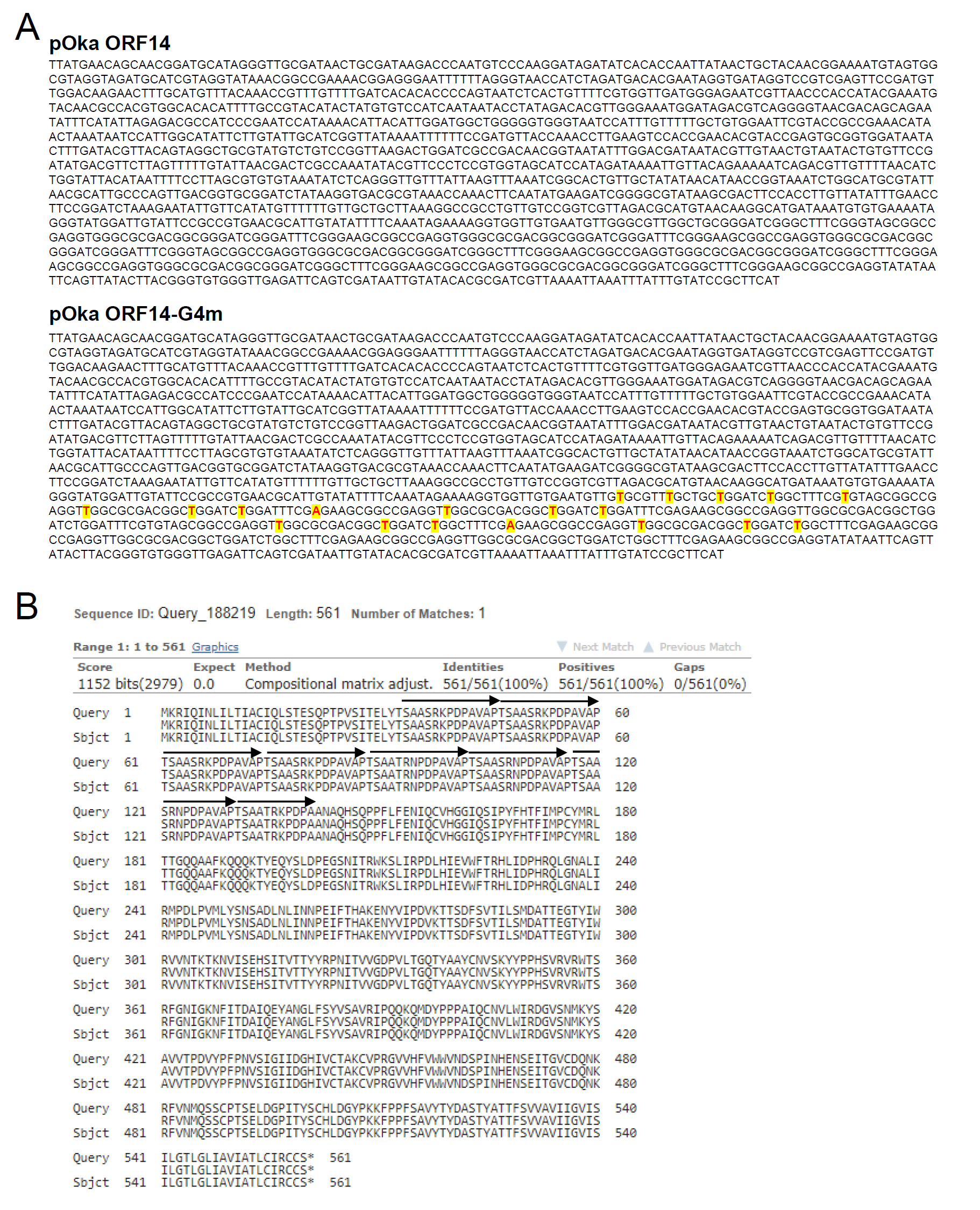

Supplement: S5 Fig — (A) The complete sequences on the template strand of the wild-type ORF14 gene of pOka and its G4-disrupted mutant (G4m) are shown. The mutated sequences in G4m are highlighted. (B) The gC amino acid sequences produced from the wild-type and G4m ORF14 genes are compared and show 100% identity. The repeated sequences of 14 amino acids encoded from the R2 region are indicated as arrows. (TIF) [file ppat.1011095.s007.tif]

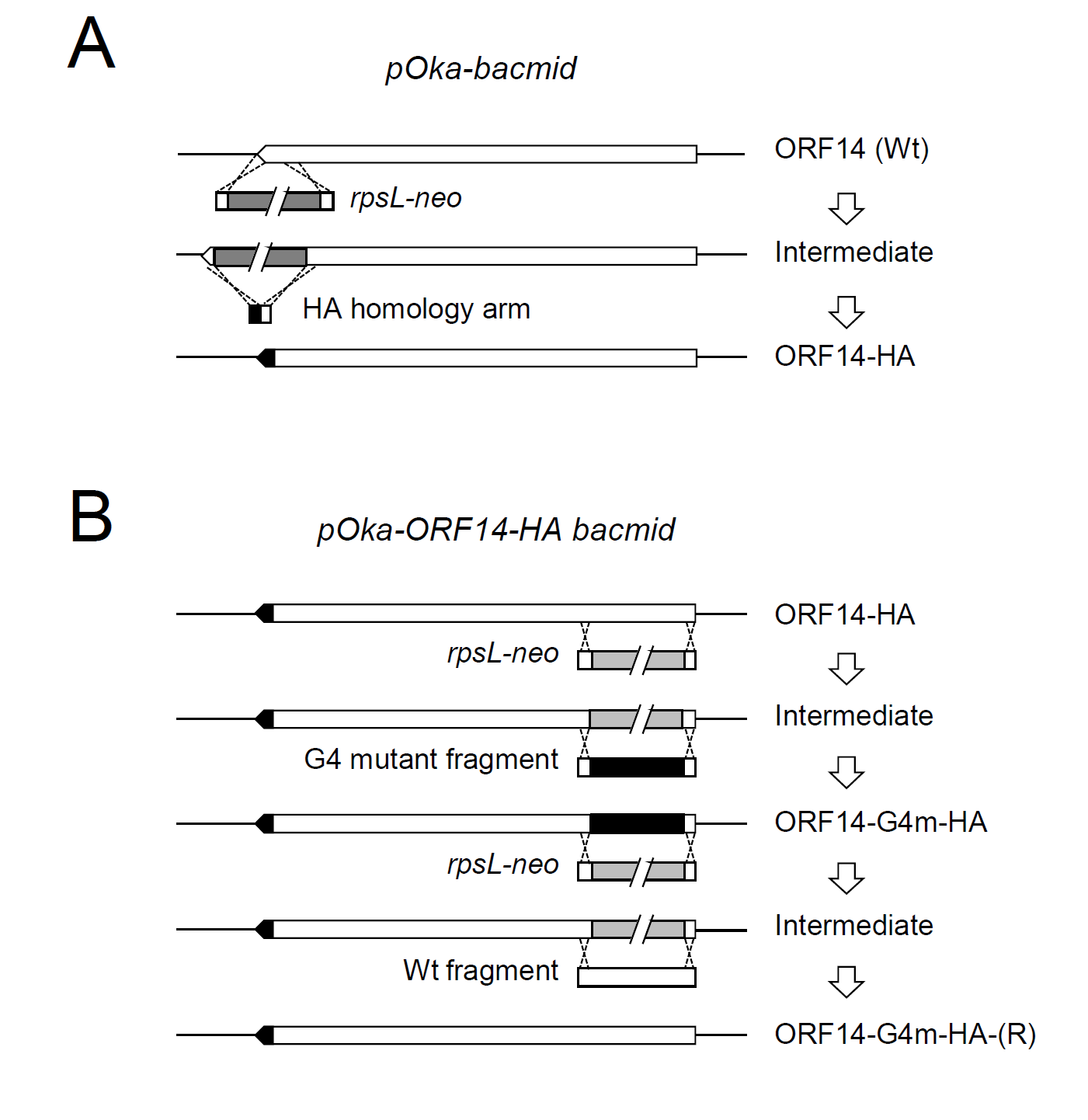

Supplement: S6 Fig — The scheme for the production of pOka bacmid containing the ORF14-HA gene (A) and pOka bacmids containing the ORF14-G4m-HA gene and its revertant gene (B). See the Materials and Methods for the detailed procedure. (TIF) [file ppat.1011095.s008.tif]

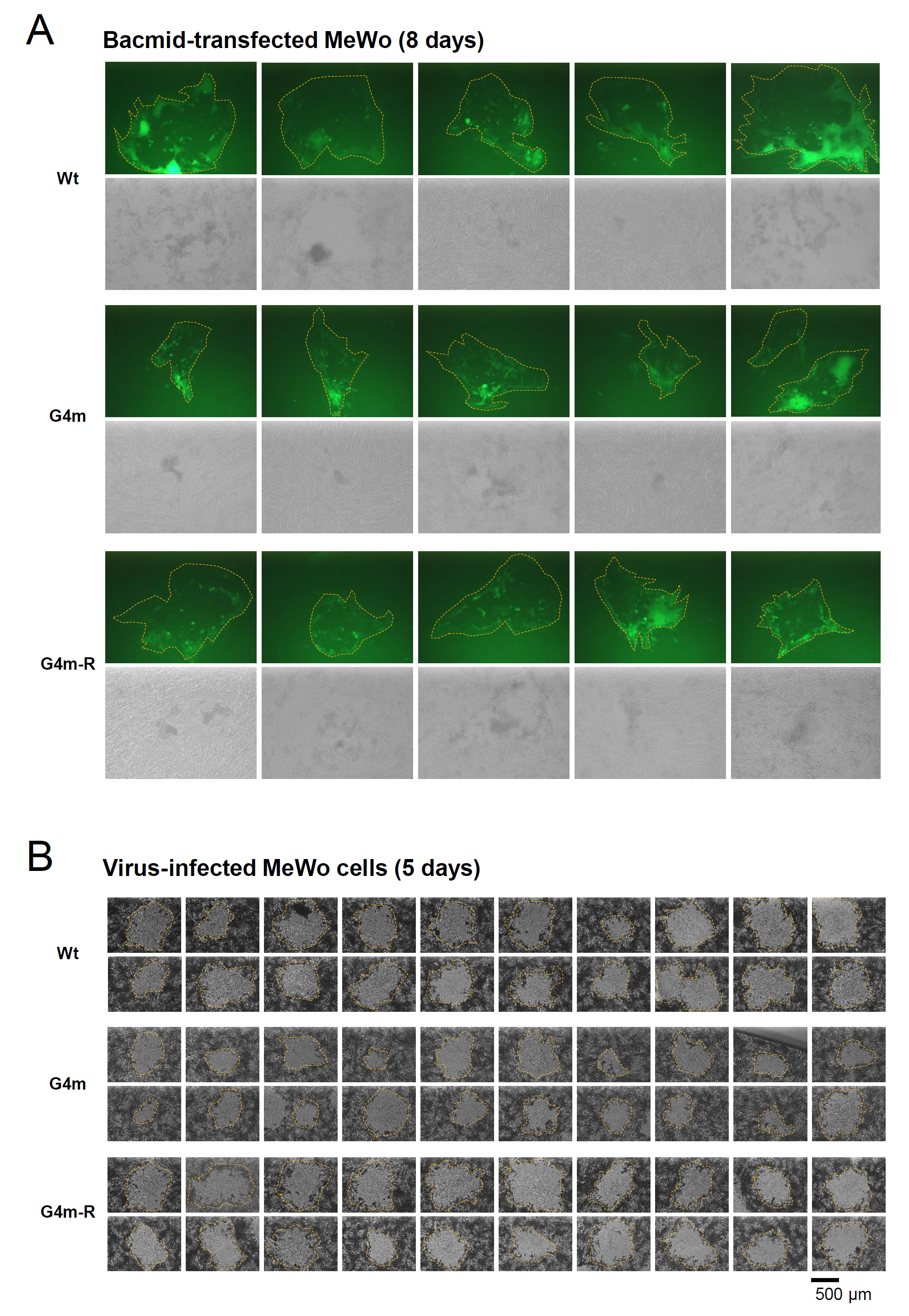

Supplement: S7 Fig — Images of MeWo cells used for measuring plaque sizes in Fig 9 are shown. The boundary of green fluorescence was used as a plaque boundary for measuring plaque regions in VZV-bacmid infected MeWo cells. (A) GFP images of cells showing plaques taken at 8 days after bacmid transfection. (B) Cell images showing plaques taken at 5 days after virus infection. To determine the plaque region in VZV-infected MeWo cells, empty regions for the confluent cell monolayer are measured. (TIF) [file ppat.1011095.s009.tif]

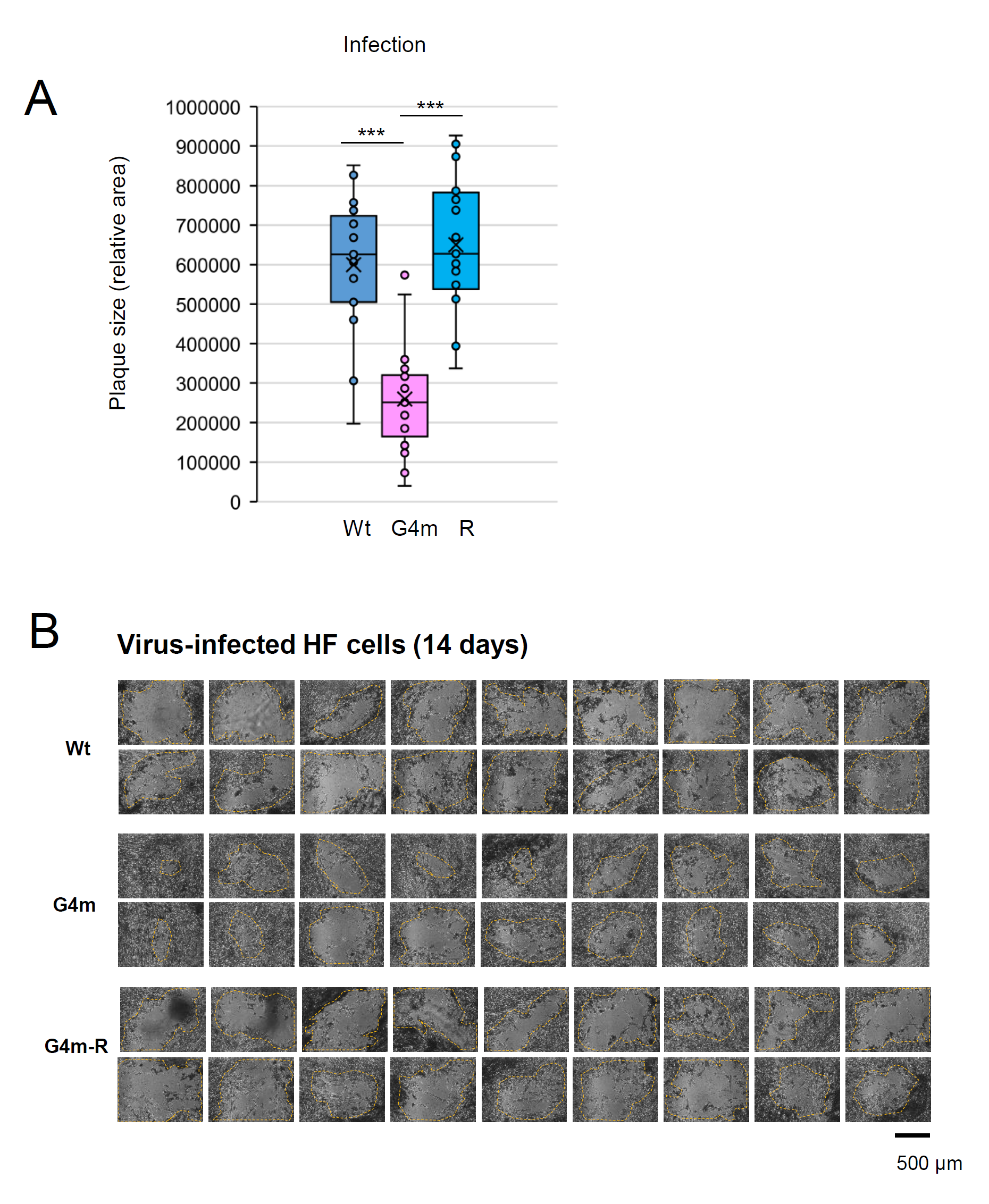

Supplement: S8 Fig — (A) HF cells in six-well plates were infected with cell-associated recombinant viruses. At 14 days after infection, cells were stained with crystal violet and plaque size was measured (n = 18). Plaque area size was measured with ImageJ in triplicate experiments and shown as a box and whisker graph. p-values <0.001 (***) are indicated. (B) Plaques images are shown as in S7 Fig. (TIF) [file ppat.1011095.s010.tif]
