# Supplementary material for: G-quadruplexes formed by Varicella-Zoster virus reiteration sequences suppress expression of glycoprotein C and regulate viral cell-to-cell spread
Source: PLoS Pathog. 2023 Jan 11;19(1):e1011095. doi: 10.1371/journal.ppat.1011095 (PMC9873165; doi:10.1371/journal.ppat.1011095)
Supplement: Attachment — Submitted filename: Review (author reponse) 2022-12-04.pdf [file ppat.1011095.s013.pdf]

Editor's comments

There is a major concern from one reviewer (#2) that the observed phenotype may be the result of the HA tag on gC interrupting proper processing, as opposed to the mutations in the G4 quadruplex. This issue, as well as the multiple comments from reviewer #1, need to be addressed.

(Response)

We thank the editor and reviewers for the helpful comments and suggestions. To address the concern for the HA tag on gC, we repeated infection assays and showed that the high MW glycosylated forms of gC-HA were expressed in ORF14-HA virus infection (new Figs 8B and D). We also performed transfection assays with ORF14 plasmids without a tag, and showed that G4-disrupting mutations increased gC transcription (new Fig 7E). We have positively addressed all comments from the reviewers as below.

Reviewer's Responses to Questions

Part I - Summary

Reviewer #1:

Chung and co-authors submitted a manuscript dealing with the presence of G-quadruplexes (G4s) in the genome of Varicella Zoster virus (VZV). In particular, the research work intends to shed light on the downregulating role of a cluster of repeated short regions, able to fold into G4s, on the expression of the viral glycoprotein C. The work is clearly written, and the topic is very interesting. Nonetheless, there are issues in the experimental design and in the described results that need to be clarified.

Reviewer #2:

In this manuscript, Chung et al report evidence to suggest that there high G/C regions of VZV include structures called G Quadruplexes. G Quad elements are known to form in single stranded DNA and RNA under some considtions to form different structures that can affect expression of RNA at the transcriptional level. They particularly focus on the VZV gC protein, which in most strains has 7/2/3 or a 42bp high G+C repeating element as part of its coding region. The work claims there is the formation of G Quadruplexes by the repeat element that regulates the transcription of gC so that, when such elements are mutated, there is a higher level of gC expression.

The work first involves scanning the genome for G Quadruplex formation elements using a program. They report the finding of about 150 potential elements. Including several found in the gC coding repeating elements. The then do a series of biophysical studies on the specific elements assessed outside of the context of their VZV environment using oligonucleotides and mutant oligonucleotides. They show subtle differences in circular Dichroism spectroscopy, Thermal difference spectra and the consequences of a known binding drug to G4 Quad pyridostatin. Which inhibits the actions (it is not clear why the drug also affects the mutant oligonucleotides despite the text indicating it does not, See Figure 7b. Whiles this shows effects on the G4 Q oligonucleotides, it is of course separated from the context of the virus, so the intepretation of these data and if it occurs in the context of the virus is difficult. While interesting, I have several major concerns. I do not think this work is of sufficient significance and asdvancement for the field for publication in the Journal Plos Pathogens

(Response)

We thank the reviewer for the helpful comments. The marginal effect of PDS on the ORF14-G4m transcription in old Fig 7B would be an indirect effect of PDS on the cellular factors. To more clearly show the inhibitory effect of PDS on gC transcription, we repeated the experiment and replaced old Fig 7B with new one showing that PDS significantly reduces the transcription of wild-type ORF14 but not G4-disrupted mutant.

We agree with the reviewer that the results obtained in the context of virus infection can be different from those found in transfection assays. To reconcile this reviewer's concern, we investigated the effect of PDS on gC transcription in virus-infected cells. Since the G4 ligands could affect the expression of several viral and cellular genes from the early stage of infection, PDS treatment reduced gC and gE transcription from both wild-type and G4-disrupted (G4m) ORF14 genes; however, the gC/gE mRNA ratio was 3-fold higher in G4m virus than in Wt and revertant viruses. Furthermore, the gC/gE mRNA ratio was decreased by PDS treatment in Wt and revertant viruses, while it was increased in G4m virus. These results demonstrate that gC transcription was

less severely suppressed by PDS in G4m virus infection than in wild-type virus infection. We have added these new results as new Fig 8F (lines 338-346).

Reviewer #3:

The manuscript by Chung et al explores the potential role of G-quadruplexes for regulating expression of glycoprotein C in Varicella Zoster virus. This is a nicely presented story, beginning with a bioinformatic evaluation of potential G quadruplexes within the VZV genome and then biophysical analyses of G-quadruplexes found within the coding region of ORF 14 (gC). From there, the investigators compared WT gC to a gC engineered with disrupted G-quadruplexes both in transient transfection experiments and then further via recombinant viruses. Overall, the evidence suggests that G4 formation within the gC coding sequences suppresses gC expression and that disruption of these motifs can impart an observable phenotype on virus growth in culture.

Part II – Major Issues: Key Experiments Required for Acceptance

Reviewer #1:

It is not clear how the authors chose the viral strains and bacmids used in the study. The strains pOKA and YC01 and YC02 were not introduced and a better description would help the reader.

(Response)

We used pOka since it is a parental strain of vOka, a licensed vaccine strain (Takahashi et al., 1974), and its bacmid is wildly used for analysis of VZV gene functions (Zhang et al., 2007). YC01 and YC02 are strains that we previously isolated from the patients of herpes zoster and chickenpox, respectively (Jeon et al., 2016). This information has been added in the text (lines 156-157 and 185-186).

The performed research on Putative G-quadruplex-forming sequences (PQS) on the genome and the choice to consider also bulged sequences and PQS with no loops is interesting: please add a summary table that categorizes the PQS.

(Response)

As requested, we have added a summary table that categorizes the G4 motifs predicted in Dumas and pOka (new Fig 1B) and another summary table for the G4 motifs in reiteration sequences in Dumas and pOka (new Fig 1C).

How many bulges per sequence were considered, were there sequences with more than one bulge?

(Response)

For consideration of G4 stability, only G4 motifs containing a single bulge were considered. This is mentioned in the text (lines 140-141).

The authors report a partial conservation analysis on VZV genomes, taking into consideration another strain. A conservation analysis performed on all VZV genomes in GenBank needs to be performed. Add also a detailed description on PQSs location in the genome (i.e. promoter, coding, UTR etc) (partial information is contained in the S1 Appendix file).

(Response)

As requested by the reviewer, we performed a conservation analysis on 141 VZV R2 sequences available in GenBank. Consistent with the result shown in Fig 2B, nucleotide variations in R2 largely occurred just downstream of G-runs in G4 motifs. This analysis has been added as new S2 Fig and S2 Appendix (lines 193-196).

For the G4 motifs found within ORFs, the information for their locations in the sense- or non-sense strand is provided in S1 Appendix. However, it is difficult to indicate the positions of G4 motifs in promoters and UTRs, since the transcription start sites and promoter regions are not precisely mapped for all ORFs. To reconcile the reviewer’s point, we analyzed the G4 motifs in IRs, which contains ORFs whose promoters and

transcription start sites have been reported. We have added the locations of G4 motifs in IRs in detail as new S1 Fig (see below).

The authors noticed that many PQSs are located in the internal and terminal repeats. Which genomic features are contained in those regions? Promoters? Enhancers? Origins of replication?
(Response)
As requested by the reviewer, we have added more analysis of G4 motifs in IRs and TRs. IRs contains ORF62, OriS, ORF63, and ORF64. TRs, which contains ORF69, ORF70, OriS, and ORF71, is an inverted sequence of IRs. The G4 motifs in IRs are found upstream of ORF61, in the sense- and non-sense strands of ORF62, and in OriS, which overlaps with the bi-directional promoter for ORF62 and ORF63. The positions of G4 motifs in IRs are indicated in new S1 Fig. We found that GQ65 lies in the minimal ORF61 promoter activated by the ORF62 transactivator, with overlapping with two non-canonical Sp1-binding sites, which are required for effective ORF61 expression (Wang et al., 2009). The ORF62/63 intergenic region containing OriS acts as a bi-directional promoter for ORF62 and ORF63 transcription (Jones et al., 2006). Several G4 motifs are found in this intergenic region. GQ94 overlaps with the promoter region of ORF62 (Moriuchi et al., 1995), while GQ103 and GQ104 are located in the promoter of ORF63 (Kost et al., 1995). Besides the R4A reiteration sequence, the OriS region contains three consensus binding sites (Boxes A, B, and C) for the VZV origin-binding ORF51 protein and a neighboring 46-bp AT-rich palindrome sequence (Hay and Ruyechan, 2007; Stow and Davison, 1986; Stow et al., 1990). Notably, GQ95 and GQ96 are found in R4A, and GQ97 and several G4 motifs overlapping with GQ97 are localized between ORF51-binding sites (Khalil et al., 2015). This information has been added in the text (lines 164-179).

The final sentence of “Distribution of G4 motifs in R2” (page 7, lines 165-166) is not clear. Figure 2b shows that mutations are on non-G nucleotides but the sentence states the opposite. Can the authors clarify what was the conveyed message?
(Response)
Mutations are on non-G nucleotides. For clarification, we have modified the sentence as “nucleotide variations found in R2 appeared to occur adjacent to G-runs in G4 motifs” (line 195).

The authors mutated the G residues (Page 7, lines 174) to T o A, but it is not explained why these nucleotides were chosen. Were all mutations aligned with the wt coding sequence (as shown in Fig S2 C), to state that the coding potential was not disrupted? Can the authors add new supplementary material on these analyses?
(Response)
We changed the G residues in G-runs to T or A but not to C to minimize the possibility of formation of unexpected secondary structures with neighboring G-rich sequences. When we made the G4-disrupted (G4m) ORF14 gene, all G4 motifs in ORF14 were replaced with the mutant sequences used for *in vitro* analysis for CD analysis (in S1 Table). Therefore, the coding potential is not changed. This has been described in the text (lines 257-258).

Page 8, line 184, the authors define the sequence with a positive peak around 280 nm and a negative peak around 245 nm as an unusual structure. Are there differences between the recorded spectra and those provided by a random nucleotide unstructured sequence?
(Response)
The peak and trough is different from that of a single-stranded 24mer-poly(T) (Ravichandran et al., PLoS Pathog. 2018). We have added this information in the text (line 215).

Figure 4 is very difficult to interpret (too many spectra on the same graph), a better one should be provided.
(Response)
We showed many spectra on the same graph to determine which spectra fit to the typical TDS patterns of G4s. To make it easier to distinguish each spectrum, we have replaced the old graph with the new one marked with finer lines in new Fig 4.

Page 8, lines 205-210, may the authors comment on the thermal stability and further G4-ligand stabilization of sequences taking into consideration the presence of bulges, short and long loops? Is there a trend in PDS binding and stabilization?

(Response)

We previously found that conventional G4 structures generally show higher thermal stability than long-loop and bulged G4 structures with or without stabilizing ligand treatment (Ravichandran et al., PLoS Pathog. 2018). The G4 structures found in R2 are long-loop or bulged G4s and their thermal stability appeared to be determined by specific sequences rather than G4 structures. We have added this information as "The thermal stability appeared to be determined by sequences rather than G4 structures." in the text (lines 239-240).

pSG5 vector is used for different studies, it is not clear which promoter drives gC expression? Are there PQS in the expressing promoter?

(Response)

In pSG5, the simian virus (SV) 40 early promoter drives gC expression. This has been described in the text (line 261). There are G4 motifs for long-looped or bulged G4s, but not conventional G4, in the SV40 promoter. However, it does not affect our interpretation of the results, since the same promoter drives both wild-type and G4-disrupted ORF14 genes.

Please add a scheme showing the mutations inserted in the gC sequence

(Response)

We have marked the mutated sequences in the ORF14-G4m gene in S4A Fig.

Figure 7 A, why do the authors perform an IP prior to WB? What are the bands around 140 KDa? If they were specific HA targets, why were they not concentrated by the IP passage? There is a band corresponding to gC-HA also in the first lane of EV (empty vector): can the authors comment on that? Panels B and C, was a normalization formula used (B-actin as reference is stated in the methods section)?

(Response)

In the previous version, we did an IP prior to WB to enrich gC-HA because its level in total cell lysates was very low. We performed transfection experiments again and prepared cell lysates with a different method (using RIPA buffer). We have indicated it in the Materials and Methods (line 486) and in the figure legend (line 952). The new result of WB now clearly shows gC-HA expression in total cell lysates (in new Fig 7A). The bands in the lane of EV were considered to be non-specific and marked as an open circle. Panels (B) and (C) show the results normalized with the mRNA level of b-actin. This normalization method is indicated in the figure legend (line 957).

Figure 8 panel b, gC protein is indicated at a different height with respect to the band shown in Fig. 7.

(Response)

As shown in new Fig 7A, we observed that the non-glycosylated form of gC-HA largely accumulated in transfected 293T cells, although some glycosylated forms were also detectable. This major form of gC-HA in transfected 293T cells was larger than that expected (~64 kDa), due to the addition of extra-amino acids (from attB2 sequence) between gC and the HA tag, which was introduced during cloning using LR clonase (Gateway technology). This information of the gC-HA plasmid has been added in the text (lines 264-268). We do not think this addition of the linker region affected gC transcription since the production of gC mRNA from the untagged ORF14 plasmid was similarly increased by G4-disrupting mutations (in new Fig 7E, lines 294-296). Recombinant viruses were designed to express gC-HA proteins without inserting additional amino acids between gC and HA. In immunoblotting with anti-HA antibody, the gC-HA proteins in ORF14-HA virus-infected cells were expressed as highly glycosylated forms with a major form between 100 and 140 kDa (new Figs 8B and D).

In the last result section, (G4 formation suppresses gC expression at the transcriptional level during virus infection) the infection assays are described in a very unclear way (page 10, lines 268-271), please carefully revise all this part.

(Response)

The sentence was modified as “Human fibroblast (HF) cells were infected with wild-type pOka (prepared from the pOka bacmid) and pOka-ORF14-HA viruses and their growth was measured by luciferase activity. The growth of the ORF14-HA virus was similar to that of the wild-type virus, indicating that the C-terminal HA tag on gC did not affect viral replication (Fig 8A). In immunoblotting with anti-HA antibody, the gC-HA proteins in ORF14-HA virus-infected cells were expressed as highly glycosylated forms with a major form between 100 and 140 kDa (Fig 8B)” (in lines 309-315).

The authors state that the virus expressing the mutated gC, which is expressed earlier and more abundantly than the wild type counterpart, attenuates the cell to cell spread in plaque reduction assays. The plaque size may depend on various factors. Please analyze and show the overall replication kinetics of the mutated virus, to understand whether the gC G4-related mutation hampers or exacerbates VZV infection. Moreover, the viral titres generated by the two viruses (wild type and mutated) would give information of the viral infectivity in human cells.

(Response)

The overall growth of the wild-type, G4-disrupted mutant, and revertant viruses was determined by measuring viral titers (PFU/ml) at the different time points after virus infection and shown as graphs (in Fig 8C). The mutant virus showed slightly reduced growth compared to the wild-type and revertant viruses (lines 320-325).

Are there circulating strains bearing the studied mutation?

(Response)

we performed a conservation analysis on 141 R2 sequences available in GenBank and found that nucleotide variations in R2 largely occurred just downstream of G-runs in G4 motifs. This analysis has been added as new S2 Fig (lines 193-196).

Reviewer #2:

They then assess the effect of mutating the g Quad elements on gene expression from a strong transfection based plasmid promoter and from a recombinant virus This raises my Major concern. In both transiently infected and recombinant infected cells, they report the expression of gC using a tagged protein. The gC protein detected in infected cells and from transiently expressed protein is far too small for fully mature gC. Multiple authors have shown the major predominant form of gC is 100-110KD, and is bigger than gE. gC contains 40KD of additional post translational modifications. However the gC in the blots in figure 8b and d, as well as in 7A, all show a protein at less than 75Kd. I do not know why, but one reason is that the C terminal HA tag is affecting gC processing; or that the mutations made are affecting translation of gC. Whatever the basis, all the subtle virus phenotypes (and they are subtle, as gC is not required for VZV growth; could all be a result of the errant processing of the protein. As such, this must be resolved for the data to be of meaning. Blots are not quantified in anyway and need to be

(Response)

Thank you for this critical comment. We repeated the experiments by employing a different cell lysis method (using RIPA buffer) to make cell lysates. We observed that gC-HA was expressed largely as a unmodified form in transfected 293T cells, although higher molecular-weight glycosylated forms were also detected (new Fig 7A). However, in recombinant virus-infected human fibroblast cells, high molecular-weight glycosylated gC-HA forms were observed, with the major band migrating between 100 and 140 kDa (new Figs 8B and D). These have been described in the text (lines 264-268 for new Fig 7A and lines 313-315 for Fig 8D).

To confirm that the gC-HA transcription in G4-disrupted (G4m) ORF14 plasmid was not affected by the addition of HA tag, we produced Wt and G4-disrupted ORF14 plasmids without a tag. We consistently found an increased transcription in G4-disrupted ORF14 (new Fig 7E, lines 294-296). This showed that the increased gC transcription of the G4-disrupted ORF14 gene is not due to the introduction of HA tag.

The gC and gE levels in virus-infected cells were quantitated with Image J and shown as graphs in new Fig 8D.

2.The blots for gC expression are poor and not easily visible. In contrast blots for gE are way overexposed in figure b and d. these need to be repeated and fully quantified
(Response)
We repeated western blot assays and replaced old Fig 8D with new one in which gC is clearly visible. The quantitation results of gC and gE bands are shown in new Fig 8D.

3. The RNA for gE is different for the mutant and the parent/repaired virus (and apparently significant) (8E) no explanation is provided.
(Response)
We repeated the infection experiments with new viral stocks. As shown in Fig 8C, the growth of the ORF14-G4m virus was slightly lower than those of Wt and revertant viruses. In new Figs 8E and F, the gC mRNA levels in G4m virus were much higher at 72 h and 96 h than those in wild-type and revertant viruses, while gE mRNA levels in G4m virus similar or reduced compared to those in wild-type and revertant viruses. The new results of gC and gE mRNA levels have been described in the text (lines 333-337).

Reviewer #3: (No Response)

Part III – Minor Issues: Editorial and Data Presentation Modifications
Reviewer #1:
In the introduction, the infection linked to VZV was classified as an early childhood, but nothing is said about the worldwide distribution of VZV-positive persons and about the results of the worldwide vaccination campaign (so the actual VZV circulation in the population). The social health burden brought about Varicella and its reactivation (shingles) should be more emphasised.
(Response)
As requested by the reviewer, we have added more information for VZV in the Introduction as "The most common complication of herpes zoster is chronic nerve pain (called postherpetic neuralgia) (Sampathkumar et al., Mayo Clin. Proc. 2009) and about 25% of immunocompetent adults older than 50 years experiences herpes zoster-related complication (Yawn et al., Mayo Clin. Proc. 2007). Although VZV vaccines can reduce varicella and herpes zoster effectively (Wang et al., Ther. Adv. Vaccines. 2016), the global number of VZV incident case has increased, with more than 83 million new VZV infection cases in 2019 (Huang et al., J. Med. Virol. 2022). Herpes zoster is a new global health burden" (lines 92-97).

What is DW? Is it the vehicle used in controls? This is not clearly stated
(Response)
DW is used as a control for ligands since PDS was dissolved in DW. It was indicated in footnotes in Table 1.

Reviewer #2:
4. A few spellers including the lack of m last lane figure 6. Also "strand" versus "stand" in a couple of places Also the references lack capitalizations and occasionally some reference details
(Response)
We have corrected those mistakes (Fig 6 and lines 53, 392, and 394)

5. Methods lack how plaques were quantified, and measured. No details of the imaging process used. Studies were done in mewo cells in which plaques are invariably asymetric
(Response)
For measurement of plaque size, images of GFP+ plaques were taken and plaque area size was measured with ImageJ. This information has been described in the legend of Fig 9 (lines 996-1002).

We have also performed plaque assays in human foreskin fibroblast cells after recombinant virus infection. This new results (in new S7 Fig) also showed a smaller plaque phenotype of the G4m virus compared to the wild-type and revertant viruses (lines 357-358).

<u>Reviewer #3:</u>

Type page 5 line 112, should be “disrupted”

(Response)

We have correct the mistake (line 118).

Fig. 7: The difference in RNA levels is about 2-fold between WT and G4m, yet protein levels seem to be substantially more than that. The investigators should provide additional explanations, addressing such issues as whether G4 might affect mRNA stability or translation efficiency to account for this. An additional consideration to address is whether WT versus G4m gC mRNA are processed and transported from the nucleus to the cytoplasm with equal efficiency.

(Response)

The difference of gC-HA levels in WT and G4m plasmid-transfected cells appeared to be more than the difference of their mRNA levels. To address whether G4s affected mRNA stability, we compared the stability of WT and G4m ORF14 mRNAs by performing qRT-PCR at the different time points after Actinomycin D treatment. The results showed that the stability of two mRNAs are similar. We have added this results as new Fig 7D and described it in the text as “The increased gC-HA mRNA transcription in the G4-disrupted ORF14 was not due to the enhanced stability of gC-HA mRNA since the stability of mRNAs produced from wild-type and G4m ORF14 was similar (Fig 7D)” (lines 288-290). We did not compare mRNA transport efficiency in transfected cells since the difference in gC protein levels between WT and G4m was similar to that in mRNA levels in virus-infected cells (see below).

Fig. 8: Within the context of the virus, mRNA levels for gC seem to be concordant with protein expression (in contrast to the transfection experiments). The investigators should provide some speculation to explain these apparent discrepancies.

(Response)

In our new results of virus-infected cells (new Figs 8D and E), the gC protein difference between Wt and G4m viruses was similar to their mRNA difference. So, the different effects of the mutations on mRNA and protein levels are observed only in transfected cells. We have described our speculation on this as “We observed that the difference in gC mRNA levels between Wt and G4m was less than that in gC protein levels in transfected cells. Although the mutations introduced did not affect mRNA stability, it cannot be excluded that they also affected protein expression at the post-transcriptional level in transfection assays.” (lines 290-294).

Some VZV strains apparently have varying numbers of 42bp repeats within their gC coding regions. Additional commentary should be provided on whether the number of G4 repeats might have additional positive/negative effects on gC expression.

(Response)

We think that ORF14 with more 42-bp repeats will be more suppressed due to G4 formation. We have added our thought in the Discussion as “Since the copy number varies in some strains, it is speculated that strains with more 42-bp elements in ORF14 will express less gC due to G4 formation and show better cell-to-cell spread” (lines 416-418).
