# Supplementary material for: G-quadruplexes formed by Varicella-Zoster virus reiteration sequences suppress expression of glycoprotein C and regulate viral cell-to-cell spread
Source: PLoS Pathog. 2023 Jan 11;19(1):e1011095. doi: 10.1371/journal.ppat.1011095 (PMC9873165; doi:10.1371/journal.ppat.1011095)
Supplement: Attachment — Submitted filename: Author response (for R2).docx [file ppat.1011095.s014.docx]

**Editor’s comments**

The reviewers are impressed with the revised manuscript and ask only for some small changes to the text. We invite you to make the changes you see fit and resubmit the manuscript for quick, editorial review.

**Reviewer Comments (if any, and for reference):**

**Part I - Summary**
Reviewer #1: Chung and co-authors submitted a revised manuscript dealing with the presence of G-quadruplexes (G4s) in the genome of the Varicella Zoster virus (VZV).
We thank the authors for considering and addressing the major issues raised in the first revision.
The information added in the introduction and the prediction and distribution of PQSs were appreciated. The initial part of the manuscript is now clearer and more complete.

Reviewer #2: the authors have done a thorough and remarkable job and have made the work far more convincing than the first version. figures have been clarified and better explained.

indeed i am ready to almost reccomend it being accepted for publication.

the article needs a spellcheck, as quite a few of the revised comments contain spellers or incorrect grammatical wordings. for a couple of examples see iines 95 global incident case

ln 169 lin 195 and other places. in s1 fig should be figure S1

line 266 the unmodified from of

(Response)

Thank you. We have changed the text as follows. Line 91: “the global number of VZV incident case” has been changed to “the number of VZV global incident case”. Lines 169 and 195: The Journal use S1 Fig instead of Fig S1. Line 266: “the unmodified from of” has been changed to “the unmodified form of”.

265 how do they know the other forms are glycosylated?

(Response)

We have modified the text as “...we observed the unmodified form of gC-HA largely accumulated in transfected 293T cells, although some modified forms (probably, glycosylated forms) were also detectable (Fig 7A)” (lines 264-266).

Reviewer #3: (No Response)

**Part II – Major Issues: Key Experiments Required for Acceptance**

Reviewer #1: none required

Reviewer #2: no mJOR ISSUES

Reviewer #3: (No Response)

**Part III – Minor Issues: Editorial and Data Presentation Modifications**

Reviewer #1: Still, there are minor issues with the new data:

- A summary table for all the analyzed PQS, reporting CD data on topology, and stability and the results of the TDS would help the reader to have an overview of all sequences and in particular of those reported with atypical CD spectra.

(Response)

We initially predicted 13 different G4 motif sequences in ORF14 and analyzed their CD patterns, but did not measure TDS and thermal stability (Tm) for all predicted G4 motifs. Therefore, it is difficult to summarize all data directly into a table. In the text, we articulated the logic used to narrow down the G4 motifs for further in vitro analysis.

- The presence of a table/figure panel reporting the aminoacidic changes caused by the G mutations would help the reader. Moreover, the authors should comment on how the changed aminoacids may influence protein folding and may contribute to the phenotypic changes of the virus. Is this new protein degraded faster than wt ORF14? which protein domain is involved in aminoacidic changes? Does mORF14 accumulate in cell granules, changing the ability of VZV to replicate? Please complete and discuss

(Response)

The G mutations we introduced to make the mutant oligos and the mutant ORF14 gene did not change amino acid sequences. It has already been described in the text as “...the G4-disrupted ORF14 mutant DNA (denoted as ORF14-G4m), in which all G4 motifs were replaced with the mutant sequences used for *in vitro* analysis (in S1 Table), was synthesized (S1 Fig). This ORF14-G4m DNA contains the same coding potential as the wild-type ORF14 (S3 Fig)” (lines 257-260). To clarify this point from the earlier part of the manuscript, we have modified the sentence in the text as “...their mutant sequences, in which three or five G-to-T or G-to-A changes are introduced in G-runs to disrupt G4 formation without affecting coding potential (S1 Table)” (line 205).

- The authors conclude that the lack of G4s changes the VZV phenotype: this information is not sustained by the experimental data. mORF14 has aminoacidic changes that may cause viral cycle changes, this aspect needs to be commented.

(Response)

The G mutations introduced to make the mutant ORF14 gene did not cause any amino acid changes (see above).

Reviewer #2: spellers and ways of saying things and Figures need to be corrected

(Response)

We have corrected typo errors.

Reviewer #3: (No Response)
